# Stratified Approaches to Antiplatelet Therapies Based on Platelet Reactivity Testing

**DOI:** 10.3389/fcvm.2019.00176

**Published:** 2019-12-03

**Authors:** Małgorzata Ostrowska, Jacek Kubica, Piotr Adamski, Aldona Kubica, Ceren Eyileten, Marek Postula, Aurel Toma, Christian Hengstenberg, Jolanta M. Siller-Matula

**Affiliations:** ^1^Department of Cardiology and Internal Medicine, Collegium Medicum, Nicolaus Copernicus University, Bydgoszcz, Poland; ^2^Department of Health Promotion, Collegium Medicum, Nicolaus Copernicus University, Bydgoszcz, Poland; ^3^Department of Experimental and Clinical Pharmacology, Centre for Preclinical Research and Technology (CEPT), Medical University of Warsaw, Warsaw, Poland; ^4^Department of Cardiology, Medical University of Vienna, Vienna, Austria

**Keywords:** P2Y_12_ inhibitors, antiplatelet therapy, ACS, HPR, LPR, precision medicine

## Abstract

Antiplatelet therapy with P2Y_12_ receptor inhibitors (clopidogrel, prasugrel, ticagrelor, cangrelor) is a cornerstone of medical therapy after percutaneous coronary interventions. Significant prevalence of high on-treatment platelet reactivity (HTPR) on clopidogrel treatment led to introduction of more potent P2Y_12_ inhibitors: prasugrel (a third generation thienopyridine), ticagrelor, and cangrelor (cyclopentyl-triazolo-pyrimidines). Nevertheless, more potent platelet inhibition and resulting low on-treatment platelet reactivity (LTPR) has led to increased risk of major bleeding events. These limitations resulted in a need for an individualized antiplatelet therapy approach. This review discusses the current role and future perspectives of diagnostic tools such as platelet function testing to optimize antiplatelet therapy with a focus on deescalating therapies to reduce bleeding risks.

## Role of Platelets in Arterial Thrombosis

**Figure 1:**
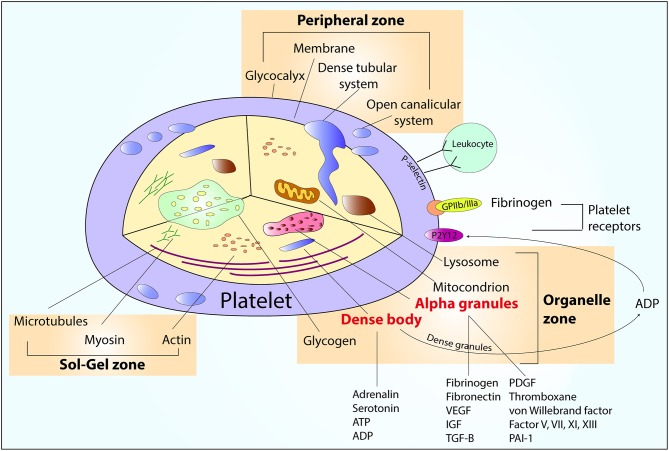
An overview of platelet structure. ADP, adenosine diphosphate; ATP, adenosine triphosphate; IGF, insulin-like growth factor; PAI-1, plasminogen activator inhibitor; PDGF, platelet-derived growth factor; TGF-β, transforming growth factor beta; VEGF, vascular endothelial growth factor.

Myocardial infarction (MI) is generally a consequence of unstable atherosclerotic plaque rupture or erosion, caused by endothelial damage (1). In patients with ST-segment elevation myocardial infarction (STEMI), the rupture of atherosclerotic plaque is associated with exposure of the lipid core and subendothelial collagen fibers, both of which initiate activation of platelets, and thrombus formation that usually lead to acute obstruction of the coronary artery (1). On the other hand, in patients with non-ST segment elevation myocardial infarction (NSTEMI) the MI is usually caused by a clot formed on unstable coronary plaque, which does not produce complete obstruction of the artery lumen (1). Excessive activation and aggregation of platelets play a pivotal role in the pathogenesis of both types of MI (2). Platelets are the smallest, anuclear morphotic elements of the blood, which derive from megakariocytes and live 7–10 days. Their surface is covered with multiple receptors and their organelle include factors promoting the clot formation (Figure 1). Platelets are responsible for the primary hemostasis, that consists of platelet adhesion, secretion, and aggregation (2). Vascular injury and exposure of the von Willebrand factor initiates platelets adhesion and activation, as a result the surface integrins α_2_β_1_ and α_2b_β_3_ (also called GP IIb/IIIa) gain high affinity to collagen and fibrinogen (3, 4). One of the most powerful modulators of platelet function is ADP, the main agonist of platelet P2Y_1_ and P2Y_12_ receptors (5). Stimulation of the P2Y_1_ receptor results in phospholipase C activation (6, 7), while stimulation of the P2Y_12_ receptor deactivates adenyl cyclase resulting in termination of cyclic adenosine monophosphate production, translating into lack of inhibition of the phospholipase C (8). Stimulation of both P2Y receptors leads to hydrolysis of phosphatidylinositol by the activated phospholipase C to triphosphate inositol and diacylglycerol (3). Triphosphate inositol is responsible for opening of the membrane calcium channels and influx of calcium, which facilitates cytoskeleton modification and change of shape to spherical, transport of α granules and dense bodies to the central part and release of their components (6). The process of aggregation is based on bridging of two neighboring platelets with fibrinogen, via activated GP IIb/IIIa platelet membrane receptors, allowing formation of the primary clot (9). Further platelet activation initiates next phase of platelet aggregation associated with cyclooxygenase-1 mediated production of thromboxane A_2_ from arachidonic acid. Thromboxane A_2_ further promotes platelet aggregation and vasoconstriction (10). The next stage is secondary hemostasis, it is initiated by platelet products and mediated by coagulation factors (2). Undesired platelet activation, leading to clot formation inside the coronary arteries explains the pathomechanism of MI and stent thrombosis—a possibly lethal complication of percutaneous coronary intervention (PCI) with stent implantation (1, 11).

## Antiplatelet Agents

Current armamentarium of antiplatelet agents includes four groups of drugs and is summarized in Table 1.

**Table 1:**
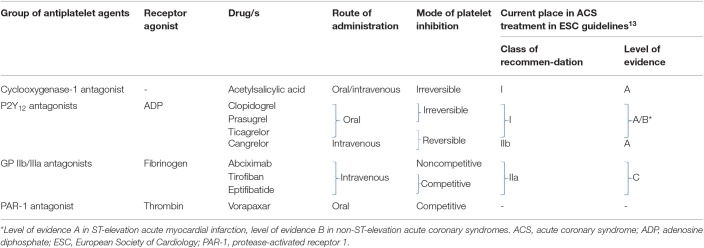
Groups of antiplatelet agents.

*Level of evidence A in ST-elevation acute myocardial infarction, level of evidence B in non-ST-elevation acute coronary syndromes. ACS, acute coronary syndrome; ADP, adenosine diphosphate; ESC, European Society of Cardiology; PAR-1, protease-activated receptor 1.

### Aspirin

Aspirin represents the cornerstone of antithrombotic therapy. Aspirin is an irreversible antagonist of the cyclooxygenase-1, that blocks the production of the thromboxane A_2_–one of the most powerful promoters of platelet aggregation (13). In patients with acute coronary syndrome (ACS), an initial oral loading dose of 150–300 mg of non-enteric coated formulation is recommended, followed by 75–100 mg once daily regimen (12, 14, 15). Life-long maintenance therapy with acetylsalicylic acid is indicated in all patients in secondary prevention of coronary artery disease (16). Although arachidonic acid induced platelet aggregation varies according to several variables (as age or sex), no routine monitoring of its treatment is required (15, 17). In case of aspirin intolerance, chronic clopidogrel therapy is recommended as an alternative (16).

### P2Y_12_ Receptor Antagonists

Dual antiplatelet therapy, composed of an aspirin and an antagonist of the platelet P2Y_12_ receptor, is a foundation of modern ACS therapy. There are three types of purinergic receptors: P2X_1_, P2Y_1_, and P2Y_12_ on the platelet surface, but only the P2Y_12_ has become a target for antithrombotic therapies that is used in everyday clinical practice (17). ADP is an agonist of the P2Y_12_ receptors. It activates the P2Y_12_ receptor via stimulation of the G_α*i*2_ protein, which deactivates adenyl cyclase translating into decreased cyclic adenosine monophosphate synthesis, which is responsible for phospholipase C inhibition, thus leading to platelet aggregation (8). Stimulation of the G_α*i*2_ protein by ADP activates also the GP IIb/IIIa receptor leading to induction of fibrinogen bridging, and initiation of the secretion of platelet derived products (18).

Contemporary armamentarium of the P2Y_12_ receptor inhibitors includes two thienopyridines: clopidogrel and prasugrel, and two non-thienopyridine drugs: ticagrelor and cangrelor (19). Thienopyridines are oral pro-drugs demanding hepatic activation via cytochrome P450, their metabolites irreversibly bind to the P2Y_12_ receptors for 7–10 days, which may impact the time to surgery after cessation (20). Whereas, both non-thienopyridines are potent, reversible and direct acting drugs, characterized by different route of administration—ticagrelor is administered orally, while cangrelor intravenously. Beside antiplatelet action, P2Y_12_ receptor inhibitors seem to exert a whole palette of pleiotropic effects including: increased adenosine plasma concentration in ticagrelor treated patients leading to increase in adenosine-related coronary blood flow, cardioprotection, promotion of the release of anticoagulative factors (21). These off-platelet effects are also associated with dyspnea and bradycardia. Additional off-target effects include improvement in peripheral arterial function and endothelial function, plaque stabilization and post-conditioning mimetic effect with cangrelor observed in animal models (22). Basic characteristic of all four P2Y_12_ receptor antagonists is presented in Table 2.

**Table 2:** Characteristics of P2Y_12_ receptor antagonists.

	**Clopidogrel**	**Prasugrel**	**Ticagrelor**	**Cangrelor**
Chemical group	Thienopyridine	Thienopyridine	Cyclopentyl -triazolo-pyrimidine	Cyclopentyl -triazolo-pyrimidine
Prodrug (requiring hepatic activation)	Yes	Yes	No	No
Loading dose	300/600 mg	60 mg	180 mg	30 μg/kg bolus
Maintenance dose	75 mg	10 mg	2 × 90 mg	4 μg/kg/min infusion
Onset of action	2–4 h	30 min	30 min	2 min
Duration of antiplatelet effect	3–10 days	5–10 days	3–4 days	1–2 h
Excretion route	Renal and biliary	Renal and feces	Biliary	Renal and feces
Recommended stop of treatment before surgery	5 days	7 days	3 days	1 h

European Society of Cardiology (ESC) guidelines clearly recommend in patients with ACS the use of more potent antiplatelet agents like ticagrelor and prasugrel, with loading doses (ticagrelor 180 mg, prasugrel 60 mg), followed by maintenance doses (ticagrelor 90 mg twice daily, prasugrel 10 mg once daily), limiting the use of clopidogrel to situations when newer agents are not available or contraindicated (600 mg loading dose, followed by 75 mg maintenance dose) (12, 14, 15). Ticagrelor can also be used in STEMI patients after fibrinolysis (23). Moreover, guidelines advise to consider the use of cangrelor in P2Y_12_ receptor inhibitor naïve patients and to continue the infusion for 2 h or until the end of PCI (12). Dual antithrombotic therapy post ACS shall be continued for up to 12 months, unless there are contraindications such as excessive risk of bleeding. Noteworthy, in high ischemic-risk patients, who have not suffered from bleeding, dual antiplatelet therapy with ticagrelor in reduced dose of 60 mg twice daily may be maintained beyond 1 year for up to 3 years (12). In patients with chronic coronary syndromes undergoing PCI, dual antithrombotic regimen composed of aspirin and clopidogrel remains the well-known standard of care, while ticagrelor or prasugrel use is limited to high-risk situations like previous stent thrombosis (16). In general, after elective stent implantation P2Y_12_ inhibitor should be continued for up to 6 months (12). Of note, in high bleeding risk patients with chronic coronary syndrome, dual antiplatelet therapy can be shortened to 1 month, while in those post ACS with high ischemic and low bleeding risk it may prolonged for up to 30 months (12, 16). A special population of interest represents patients who require combination of antiplatelet therapy and anticoagulation. Recently, it has been shown that among patients with atrial fibrillation and chronic coronary syndrome (>1-year after the index event), the addition of antiplatelet drugs, as a monotherapy or dual antiplatelet therapy, does not provide added protection against coronary events, but increases the risk of major bleeding (24).

### Glycoprotein IIb/IIIa Receptor Antagonists

Another group of antithrombotic agents are inhibitors of the GP IIb/IIIa receptors. These glycoproteins belong to adhesion molecules and are the most abundant platelet surface receptors. Their role in platelet aggregation is pivotal, after activation, and change of conformation they gain high affinity for fibrinogen, von Willebrand factor and prothrombin (25). Fibrinogen molecule has binding sites on both sides allowing bridging between two neighboring platelets, thus initiating aggregation. In everyday clinical practice we use three intravenous GP IIb/IIIa inhibitors: abciximab, eptifibatide, and tirofiban. Beside potent antiplatelet effect they can exert some off-target actions, mainly anti-inflammatory (26). The use of GP IIb/IIIa antagonists should be reserved for bail-out situations, if there is evidence of no-reflow or a thrombotic complication (class of recommendation IIa, level of evidence C) (12). Their use in patients in whom coronary anatomy is not known is not recommended (class of recommendation III, level of evidence A) (12).

## Assessment of Platelet Inhibition Under Antiplatelet Therapies

### Platelet Function Testing

Many different methods to assess platelet function exist, beginning with the historic golden standard—light transmission aggregometry, that measures the difference between light transmission through platelet rich plasma and platelet poor plasma, assessment of platelet aggregation on fibrinogen-coated microparticles (VerifyNow assay) or metal electrodes (Multiplate analyzer), measurement of the VASP protein phosphorylation (VASP assay), assessment of platelet aggregation *in vitro* in conditions similar to physiological blood flow (PFA-100, PFA-200, Innovance P2Y_12_, IMPACT-R), assessment of the clot strength (thromboelastography), measurement of the thrombocytes number before and after the addition of an agonist (Plateletworks) (27–29). It has to be acknowledged that due to great differences in assessment of platelet reactivity between available tests, a diagnosis of either HTPR or LTPR based on one method can be unconfirmed with the use of a different method. According to both American and European groups of experts there are three recommended platelet function tests: the VerifyNow assay, the Multiplate analyzer, and the VASP assay for clinical guidance (30, 31). In the HARMONIC study platelet reactivity values assessed with all three recommended platelet function tests in MI patients treated with ticagrelor correlated well with each other, however a significantly higher correlation was demonstrated between the VerifyNow and Multiplate tests than in other assay combinations (32). Interestingly, emerging concepts as platelet redox assessment (intracellular concentration of reactive oxygen species, activity of antioxidant enzymes, reduced/oxidized glutathione ratio, level of lipid peroxidation, Cu/Zn ratio, and molecular oxygen consumption) might be potentially useful to establish the platelet-related etiological factors in different disorders and to evaluate the antiplatelet therapies (33).

### High On-Treatment Platelet Reactivity (HTPR)

Numerous studies have shown that up to 40% of patients exhibit HTPR under clopidogrel treatment (34–42). There are many potential causes of this phenomenon including clinical variables such as ACS at admission, diabetes mellitus, renal failure, drug-drug interactions, non-adherence to therapy, genetic polymorphism of genes coding cytochrome P450 enzymes (crucial in clopidogrel bioactivation) or glycoprotein P (responsible for clopidogrel absorption in intestines) (37, 43–48). Recently, an association between the circulating proprotein convertase subtilisin/kexin type 9 (PCSK9) levels, HTPR and ischemic events in ACS patients undergoing PCI were described (49). There is a clear evidence showing that HTPR on clopidogrel is a significant risk factor for atherothrombotic events, including MI, stent thrombosis, cardiovascular death and cerebrovascular events (40, 50–52). There are some therapeutic options to overcome HTPR on clopidogrel. HTPR may also affect patients treated with newer, more potent antithrombotic agents such as prasugrel or ticagrelor, mainly within the first hours post loading dose in ACS patients undergoing PCI, when sufficient antiplatelet blockade is particularly desired (52–56). However, in a recently published systematic review and meta-analysis, early (>2 h pre-PCI) vs. late (<2 h pre-PCI or post-PCI) administration of loading doses of potent antiplatelet agents did not improve ischemic outcomes in more than 60,000 patients, questioning the importance of early loading (57). In contrast, early clopidogrel loading in ACS or STEMI patients reduced the risk of adverse events (57). The prevalence of HTPR in patients treated with ticagrelor was significantly lower as compared with those receiving prasugrel in a meta-analysis by Lemesle et al. (58). It was previously documented that age, gender, food, preloading with clopidogrel or genetic polymorphisms do not affect ticagrelor metabolism or its antiplatelet effect (59–61). Diversely, morphine which used to be a golden standard of care for all patients presenting with acute MI, was found to attenuate ticagrelor bioavailability and its antiplatelet action, mainly due to vomiting and decelerating the intestinal passage and absorption of ticagrelor (53, 62). There are few disputed strategies to overcome the morphine-ticagrelor interaction, either by crushing ticagrelor tablets, giving other analgesic, co-administering naloxone or metoclopramide (62–64). In a prospective, observational PINPOINT trial it has been found that ticagrelor concentration was reduced and antiplatelet response was delayed in the initial hours of treatment in STEMI patients as compared with NSTEMI patients (65). In a subsequent analysis, it has been reported that the main determinants of HTPR at 1 and 2 h after ticagrelor loading dose are presence of STEMI and morphine co-administration (66). Furthermore, the presence of STEMI and diabetes mellitus were found to be associated with impaired metabolism of ticagrelor within first 6 h post ticagrelor loading dose in ACS patients (67). It has been recently published, that bioavailability of ticagrelor in MI patients managed with mild therapeutic hypothermia after out-of-hospital cardiac arrest is significantly decreased, thus increasing the risk of stent thrombosis, a possibly lethal complication, which is not uncommon in this specific subset of patients (68, 69). The main reasons of insufficient antiplatelet effect of the P2Y_12_ inhibitors in out-of-hospital cardiac arrest survivors treated with mild therapeutic hypothermia are probably impaired gastrointestinal absorption and altered cytochrome activity causing a delay in drug metabolism (69–71). The temporary use of cangrelor may be a solution to overcome HTPR while oral antiplatelet agents start to work in resuscitated patients undergoing mild therapeutic hypothermia (72). A single dose of intravenous morphine in STEMI patients was associated with a delay in the onset of prasugrel action (73), 65% of critically ill patients display HTPR on prasugrel, mainly due to poor absorption from gastrointestinal tract, as well as increased platelet reactivity induced by generalized inflammation (74).

### Conclusion of the Chapter

- HTPR is a significant and modifiable risk factor for cardiac ischemic events and it is present frequently in clopidogrel treated patients.
- Patients treated with prasugrel and ticagrelor can display HTPR mainly in the acute phase of treatment, which can be in part related to opioid use.
- The routine use of platelet function testing to detect HTPR and undertake action is not recommended by the ESC guidelines. Nevertheless, HTPR should be taken into account, if de-escalation is undertaken from potent P2Y_12_ inhibitors to clopidogrel (12).
- HTPR can be detected by a variety of platelet reactivity testing.

### Low On-Treatment Platelet Reactivity (LTPR)

With the introduction of more potent antiplatelet agents the problem of LTPR associated with elevated bleeding risk became a major concern. In the TRITON-TIMI 38 trial the use of prasugrel as compared with clopidogrel was associated with significant increase of non-coronary artery bypass grafting related major bleeding, as well as life-threatening bleeding and bleeding leading to death according to the Thrombolysis in Myocardial Infarction (TIMI) criteria (75). While in the PLATO trial the administration of ticagrelor as compared with clopidogrel carried similar risk of major bleeding according to the PLATO trial criteria. However non-coronary artery bypass grafting related major bleeding and both major and minor bleeding occurred more frequently in the ticagrelor group (76).

Data on LTPR and bleeding on clopidogrel therapy comes from few small studies adapting different bleeding scales. Another limitation is low amount of serious bleeding events in those trials and predominant inclusion of low risk stable patients. First study reporting a link between LTPR and bleeding was conducted in 597 ACS patients treated with clopidogrel (77). In a 1-month observation period there were 16 bleeding episodes (5 serious and 11 small). Patients suffering from bleeding events were characterized by stronger platelet inhibition measured with the light transmission aggregometry (a previous golden standard) or the VASP assay. In a study by Sibbing et al. LTPR on clopidogrel (the cut-off value was based on the ROC curve analysis accounting 18.8 U according to the Multiplate analyzer) affected 39% of 2,533 patients with stable coronary artery disease undergoing PCI. Furthermore, those with LTPR had significantly higher risk of major in-hospital bleeding according to the TIMI criteria (78). Another study including 246 stable coronary artery disease patients receiving clopidogrel showed a relationship between >50% platelet inhibition measured with the light transmission aggregometry and occurrence of any bleeding event assessed with the use of very liberal bleeding scale called the BleedScore: 88% of all included bleeding events were superficial bleeding (79). Importantly, older age and female sex are important predictors of LTPR and of bleeding odds (80, 81).

There are few prospective studies providing a head-to-head comparison of platelet reactivity and bleeding risk in patients on prasugrel vs. ticagrelor (55, 82, 83). The first randomized trial included only 96 ACS patients treated with PCI (82). The half of them received ticagrelor, the other half received prasugrel, and platelet reactivity measurements were performed after 1 month. LTPR was defined as PRI ≤ 20% in the VASP assay and occurred in 58% of ticagrelor recipients and 33% of prasugrel recipients with a lack of relationship between LTPR and bleeding events. Another prospective registry including 512 patients with ACS treated with PCI (278 on ticagrelor, 234 on prasugrel) has shown that patients treated with ticagrelor were characterized by lower platelet reactivity assessed with the use of the VerifyNow device at 1 month post PCI, as compared with prasugrel (33.3 Platelet Reactivity Units (PRU) vs. 84.6 PRU; *p* < 0.001) (83). Grade 1 Bleeding Academic Research Consortium (BARC) bleeding events were more frequent in the ticagrelor arm, while grade ≥2 BARC bleeding events rate was similar irrespective of antiplatelet agent used. Another observational study assessing the relationship between occurrence of clinical events and platelet reactivity in 226 ACS patients (105 on ticagrelor and 121 on prasugrel) (55) indicated that bleeding episodes occurred in patients with platelet reactivity values ≤ 23 U as assessed with the Multiplate Analyzer.

### Conclusion of the Chapter

- Due to the widespread use of potent P2Y_12_ inhibitors, the LTPR phenotype is frequent.
- LTPR is a well-documented risk factor for bleeding complications. Platelet function guided dose-adjustment of potent P2Y_12_ inhibitors may be a potential solution in patients who are presenting with a bleeding event (12).

### Therapeutic Window Strategy

Based on the growing body of evidence showing an association between HTPR and ischemic events, and LTPR with bleeding events, the therapeutic window hypothesis was developed. It suggests that patients with platelet reactivity values within the middle range achieve the best net clinical benefit (28, 84). According to the European group of experts, the cut-off values for HTPR are as following: the VerifyNow assay >208 PRU, the Multiplate analyzer >46 Units (U) and the VASP assay >50% Platelet Reactivity Index (PRI) (31). The cut-off values for LTPR are as following: the VerifyNow assay <95 PRU, the Multiplate analyzer <19 U and the VASP assay <16% PRI.

### Conclusion of the Chapter

- The therapeutic window strategy to guide antiplatelet therapy might be an attractive strategy to improve patients net clinical benefit in terms of precision medicine.
- Clinical randomized trials aiming to answer that question are missing yet.

## Studies Investigating Individualized Antiplatelet Treatment to Overcome HTPR (Table 3)

First small randomized trials comparing individualized antiplatelet therapy vs. standard of care antithrombotic treatment showed favorable results of antithrombotic adjusted therapy with either higher clopidogrel dose or addition of GP IIb/IIIa antagonist (104, 106, 107, 110, 113, 114). First large randomized trial that has brought huge disappointment to advocates of the individualized approach was the Gauging Responsiveness with a VerifyNow assay, Impact on Thrombosis and Safety (GRAVITAS) trial showing no benefit of administration of higher clopidogrel dose vs. standard clopidogrel dosing in 2200 low-to-moderate cardiovascular risk patients undergoing PCI with HTPR on-clopidogrel when it comes to death from cardiovascular causes, MI or stent thrombosis (hazard ratio [HR] 1.01; 95% confidence interval [CI] 0.58–1.76; *P* = 0.97) in a 6 month follow-up (96). The next negative, similar size study—The Assessment by a Double Randomization of a Conventional Antiplatelet Strategy for Drug-Eluting Stent Implantation and of Treatment Interruption vs. Continuation 1 Year after Stenting (ARCTIC) trial demonstrated that the addition of acetylsalicylic acid, clopidogrel or switch to prasugrel as compared with conventional approach did not show any significant differences in the occurrence of the primary end point composed of death from cardiovascular causes, MI, stent thrombosis, urgent revascularization or stroke (HR 1.13; 95% CI 0.98–1.29; *P* = 0.10) 1 year after stent implantation in a group of 2,440 low-to-moderate cardiovascular risk patients (95). The third large trial that was prematurely ended and almost entombed the individualized approach was The Testing Platelet Reactivity in Patients Undergoing Elective Stent Placement on Clopidogrel to Guide Alternative Therapy with Prasugrel (TRIGGER-PCI). Trial included only 423 low cardiovascular risk patients with stable coronary artery disease undergoing elective PCI, and the strategy of switch from clopidogrel to prasugrel in those with HTPR on-clopidogrel did not bring reduction in the primary endpoint composed of death from cardiovascular causes and MI with concomitant increase in TIMI major bleeding at 6 months (94).

**Table 3:** Studies investigating individualized antiplatelet treatment.

**Study author/acronym**	**Population**	***n***	**Follow-up**	**Outcome**	**Method**	**Cut-off value**	**Study type**
ELECTRA (85)	MI	52	14 days	Level of platelet inhibition	VASP assay and MEA	16% for LTPR, 50% for HTPR; 19 U for LTPR 46 U for HTPR	CRT: ticagrelor standard maintenance dose 2 × 90 mg vs. ticagrelor reduced maintenance dose 2 × 60 mg in patients 30 days post MI
TOPIC (86)	PCI for ACS	646	1 year	MACE, BARC ≥2 bleeding	n/a	n/a	CRT: continuation of ticagrelor/prasugrel vs. switch to clopidogrel in patients 1 month post ACS
TROPICAL-ACS (87)	PCI for ACS	2,610	1 year	Net clinical benefit:MACE or BARC ≥2 bleeding	MEA	46 U	CRT: guided de-escalation: 7 days prasugrel 5 or 10 mg + 7 days clopidogrel 75 mg after 14 days if HTPR prasugrel 5 or 10 mg, if no HTPR clopidogrel 75 mg vs. non-guided prasugrel 5 or 10 mg
ANTARCTIC (88)	PCI for ACS	877	1 year	ST, MACE, BARC major bleeding	VerifyNow	208PRU for HTPR 85 for LTPR	CRT: guided: in case of HTPR on prasugrel 5 mg swtich to prasugrel 10 mg, in case of LTPR on prasugrel 5 mg switch to clopidogrel 75 mg vs. non-guided prasugrel 5 mg
PECS REGISTRY (89)	ACS+PCI	741	1 year	ST, MACE, BARC major bleeding	MEA	46 U	Observational: 600/150 mg clopidogrel vs. prasugel in patients with HTPR
IDEAL-PCI (90)	PCI	1,008	1 month	ST	MEA	50 U	Observational: non-HTPR on clopidogrel arm vs. HTPR on clopidogrel individualized approach (reloading with clopidogrel, ticagrelor, or prasugrel; re-testing)
ISAR-HPR (91)	PCI for CAD or ACS	999	1 month	ST, MACE, TIMI bleeding	MEA	468AUxmin	Retrospective HTPR on clopidogrel arm vs. prospective HTPR on clopidogrel individualized approach arm (reloading with clopidogrel, switch to prasugrel, re-testing)
MADONNA (92)	PCI	798	1 month	ST, MACE, TIMI major bleeding	MEA	50 U	Non-randomized, controlled: non-guided vs. guided group (up to 4 loadings with 600 mg clopidogrel or 1 loading with prasugrel in patients with HTPR)
Kozinski et al. (93)	ACS+PCI	71	1 month	Level of platelet inhibition	VASP assay	50%	Parallel-group, open-label study: patients with HTPR were assigned to prasugrel (30 mg loading dose, 10 mg maintenance dose) or clopidogrel (150 mg maintenance dose for 6 days and thereafter 75 mg maintenance dose)
TRIGGER-PCI (94)	Elective PCI	423	6 months	MACE, bleeding	VerifyNow	208PRU	CRT: prasugrel (loading of 60 mg and maintenance 10 mg) vs. clopidogrel (maintenance 75 mg) in patients with HTPR
ARCTIC (95)	PCI with DES	2,440	1 year	MACE	VerifyNow	235PRU	CRT: guided: clopidogrel (600 mg reloading and 75 mg or 150 mg maintenance) or prasugrel (60 mg loading and 10 mg maintenance) or GP IIb/IIIa inhibitors vs. non-guided: clopidogrel (maintenance 75 mg) in patients with HTPR
GRAVITAS (96)	PCI for CAD or NSTE-ACS	2,214	6 months	MACE	VerifyNow	230PRU	CRT: 300/75 mg clopidogrel vs. 600/75 mg clopidogrel in patients with HTPR
Alexopoulos et al. (97)	CAD with clopidogrel treatment	31	1 month	Level of platelet inhibition	VerifyNow	235PRU	Randomized, crossover: 10 m prasugrel vs. 150 mg clopidogrel in patients with htpr
Alexopolus et al. (98)	HD with clopidogrel treatment	21	1 month	Level of platelet inhibition	VerifyNow	235PRU	Randomized, crossover: 10 m prasugrel vs. 150 mg clopidogrel c
Capranzano et al. (99)	Clopidogrel treatment + age >75	100		Level of platelet inhibition	VerifyNow	230PRU	Observational: prasugrel in patients with htpr
Ferreiro et al. (100)	DMII	30		Level of platelet inhibition	VASP assay	50%	Observational: cilostazol vs. 150 mg clopidogrel in patients with HTPR
BOCLA Plan (101)	PCI	504		Level of platelet inhibition	IA	5 Ω	Observational: 600/150 mg clopidogrel vs. ticlopidine vs. prasugrel in patients with HTPR
Gurbel et al. (102)	Stable CAD + previous PCI	20	7 days	level of platelet inhibition	LTA	43%	Observational: single dose elinogrel 60 mg in patients with HTPR
RESPOND (103)	Stable CAD + clopidogrel	41	1 month	Level of platelet inhibition	LTA	43%	CRT crossover: ticagrelor 180/90 mg vs. clopidogrel 600/75 mg
Valgimigli et al. (104)	Elective PCI	263	In hospital	MACE	VerifyNow	235PRU	CRT: tirofiban vs. placebo in patients with HTPR
ACCEL-RESISTANCE (105)	PCI	60	1 month	Level of platelet inhibition	LTA	50%	CRT: adjunctive cilostazol vs. 150 mg clopidogrel in patients with HTPR
Bonello et al. (106)	PCI	429	1 month	MACE, ST, bleeding	VASP assay	50%	CRT: guided (repeated loading with clopidogrel 600 mg) vs. non-guided group
Bonello et al. (107)	PCI	162	1 month	MACE	VASP assay	50%	CRT: guided (repeated loading with clopidogrel 600 mg) vs. non-guided group
VASP-02 (108)	Elective PCI	153	1 month	MACE, level of platelet inhibition	VASP assay	69%	Observational: 150 mg clopidogrel in patients with HTPR
Trenk et al. (109)	Elective PCI	117	14 days	Level of platelet inhibition	LTA	14%	Observational: 150 mg clopidogrel vs. control in patients with HTPR
Cuisset et al. (110)	Elective PCI	149	1 month	MACE	LTA	70%	CRT: GP IIb/IIIa antagonists vs. control in patients with HTPR
Matezky et al. (111)	MI	200	10 weeks	Level of platelet inhibition	LTA	80%	Observational: 600/150 mg clopidogrel in patients with HTPR
Neubauer et al. (112)	Elective PCI	161		Level of platelet inhibition	IA	5 Ω	Observational: 600/150 mg clopidogrel vs. ticlopidine in patients with HTPR

*ACS, acute coronary syndrome; BARC, Bleeding Academic Research Consortium; CAD, coronary artery disease; CRT, controlled randomized trial; CYP, cytochrome P450; DM, diabetes mellitus; HD, haemodialysis; HTPR, high on-treatment platelet reactivity; IA, impedance aggregometry; LTA, light transmission aggregometry; LTPR, low on-treatment platelet reactivity; MACE, major adverse cardiovascular event; MEA, multiple electrode aggregometry; MI, myocardial infarction; n/a, not applicable; NSTE-ACS, non ST-elevation acute coronary syndrome; PCI, percutaneous coronary intervention; PRU, platelet reactivity unit; ST, stent thrombosis; TIMI, thrombolysis in myocardial infarction; U, unit; VASP, vasodilator stimulated phosphoprotein*.

More promising results on the conception of individualized approach were shown by some prospective registries (89, 90, 92). The MADONNA registry, which included 798 patients (more than one third of them had MI), has shown that the non-guided group had significantly higher risk of stent thrombosis (odds ratio [OR] 7.9; 95% CI 1.08–69.2; *p* = 0.048) at 30 days as compared with individualized therapy group (92). In the IDEAL-PCI registry the main strategy to overcome HTPR was a switch to a more potent antithrombotic agent (mainly prasugrel). At 30 days, there was only one definite stent thrombosis in the non-guided group (90). In the PECS registry, including only ACS patients, those with HTPR received either higher clopidogrel dose or prasugrel, while those below HTPR threshold received conventional clopidogrel therapy (89). The primary endpoint composed of all-cause death, MI, stent thrombosis or stroke at 1 year occurred more frequently in patients treated with higher clopidogrel doses than in conventional treatment group (HR 2.27; 95% CI 1.45–3.55; *p* < 0.0001), while the risk of ischemic events in prasugrel recipients was similar to conventional treatment arm (HR 0.90; 95% CI 0.44–1.81; *p* = 0.76). Worth adding is the fact that bleeding events (3/5 according to the BARC scale) were also more frequent in the higher clopidogrel dose group vs. conventional group (HR 2.09; 95% CI 1.05–4.17; *p* = 0.04), while in prasugrel recipients it was similar to conventional treatment arm (HR: 1.90; 95% CI 1.17–3.08; *p* = 0.01).

After failure of the first randomized trials investigating individualized antithrombotic therapy and some favorable data from registries, long-awaited results of the Platelet function monitoring to adjust antiplatelet therapy in elderly patients stented for an acute coronary syndrome (ANTARCTIC) randomized study were recently published (88). The study was designed for elderly population including patients over 75 years old undergoing PCI for ACS. Participants were divided into two groups. In the monitoring group, patients received prasugrel 5 mg daily with dose or drug adjustment in case of HTPR, while in conventional group patients were treated with prasugrel 5 mg daily. Platelet function was tested with the VerifyNow assay. The cutoff values for HTPR and LTPR were based on the American consensus of experts, accounting ≥208 for ischemic events and ≤ 85 for bleeding events (30). The primary endpoint composed of cardiovascular death, myocardial infarction, stroke, stent thrombosis, urgent revascularization, and BARC-defined bleeding complications (types 2, 3, or 5) occurred in 120 (28%) patients in the monitoring group vs. 123 (28%) in the conventional group (HR 1.003, 95% CI 0.78–1.29; *p* = 0.98). Rates of bleeding events did not differ significantly between groups. Drug or dose adjustment based on platelet reactivity measurements did not improve the clinical outcome in a group of elderly patients undergoing PCI for ACS.

### Conclusion of the Chapter

- Trials on individualized antiplatelet approach had many limitations. These concerns are mainly due to the chosen low cardiovascular risk populations (mostly stable coronary disease patients), use of different cut-off points for HTPR, predominant use of higher clopidogrel doses instead of more potent antiplatelet agents to overcome HTPR, only single switch to other dose or antiplatelet agent, delayed time of randomization (after PCI or even day after PCI) and chosen compounds of the primary endpoint (34).
- Real life data from the registries showed more promising results.

## Studies Investigating de-Escalation of antiplatelet Treatment

### TROPICAL

The randomized trial Testing Responsiveness to Platelet Inhibition on Chronic Antiplatelet Treatment for Acute Coronary Syndromes (TROPICAL-ACS) assessed guided de-escalation of antiplatelet treatment in patients with MI treated with PCI in 2,610 patients (87). Investigators of the TROPICAL-ACS trial aimed to test safety and efficacy of antithrombotic treatment de-escalation from prasugrel in the acute phase of ACS to clopidogrel in the chronic phase based on platelet reactivity measured with the Multiplate analyzer. 1,304 patients were included to the de-escalation study arm. Participants were treated with prasugrel for a week, and then switched to clopidogrel for a week and after 14 days platelet reactivity assessment was performed resulting in either continuation of clopidogrel therapy or in case of HTPR switch back to prasugrel. In the conventional study arm, 1,306 patients were treated with prasugrel for 12 months. The primary endpoint was the net clinical benefit (cardiovascular death, MI, stroke or bleeding grade 2 or higher according to BARC criteria) and it occurred in 95 patients (7%) in the guided de-escalation group and in 118 patients (9%) in the control group (p_non−inferiority_ = 0.0004; HR 0.81; 95% CI 0.62–1.06; p_superiority_ = 0.12). Despite early de-escalation, there was no increase in the primary endpoint of ischemic events in the de-escalation group (32 patients [3%]) vs. the control group (42 patients [3%]; p_non−inferiority_ = 0.0115), with similar frequency of BARC 2 or higher bleeding events in the de-escalation group vs. control group (64 [5%] vs. 79 [6%]; HR 0.82; 95% CI 0.59–1.13; *p* = 0.23). It is worth underlining that the trial was designed to test the non-inferiority hypothesis and the analysis was intention to treat. As a consequence, the platelet reactivity-guided antithrombotic drug de-escalation was non-inferior to recommended conventional 12 months prasugrel therapy at 1 year after PCI in MI patients in terms of the net clinical benefit.

### TOPIC

In the TOPIC (timing of platelet inhibition after acute coronary syndrome) randomized study, 645 patients 1 month after ACS were randomly assigned to either continuation of dual antiplatelet therapy composed of aspirin and potent antiplatelet agent or de-escalation to aspirin and clopidogrel (86). Drug de-escalation occurred without platelet function testing, however all patients underwent platelet reactivity assessment with the use the VASP assay at the time of randomization. The primary endpoint combining cardiovascular death, urgent revascularization, stroke and bleeding as defined as BARC ≥2 occurred in 85 (26.3%) patients in the unchanged drug group vs. 43 (13.4%) patients in the de-escalation group (HR 95%CI 0.48 (0.34–0.68; *p* < 0.01), with significant reduction in the occurrence of BARC ≥2 bleeding (48 [14.9%] vs. 13 [4%]; HR 95%CI 0.30 (0.18–0.50), *p* < 0.01). Additionally, the subanalysis revealed that at the time of randomization based on the results of platelet function testing, 47% of patients were classified with LTPR. Among this subpopulation, drug de-escalation brought the most prominent reduction in the primary endpoint incidence as compared with continued potent antiplatelet regimen (HR 0.29; 95% CI 0.17–0.51; *p* < 0.01). Nevertheless, the reduction of bleedings in the de-escalation group was mainly due to TIMI minimal and minor bleedings, with no difference in the major bleeding events.

### ELECTRA

In the recently published Effectiveness of LowEr maintenanCe dose of TicagRelor early After myocardial infarction (ELECTRA) study, the antiplatelet efficacy of two ticagrelor maintenance dose regimens (reduced dose of 60 mg twice daily vs. standard dose of 90 mg twice daily) in stable patients at 30 days after acute MI were compared (85). The trial included 52 patients randomized in 1:1 ratio to the reduced or standard ticagrelor maintenance dose. Platelet function testing with the use of the VASP assay and the Multiplate analyzer were performed 2 weeks after the treatment initiation. There were no significant differences in platelet reactivity between patients treated with reduced vs. standard ticagrelor dose (VASP: 10.4 [5.6–22.2] vs. 14.1 [9.4–22.1] %PRI; *p* = 0.30; Multiplate: 30.0 [24.0–39.0] vs. 26.5 [22.0–35.0] U; *p* = 0.26). Importantly, the percentage of patients with HTPR was similar in reduced vs. standard ticagrelor dose (VASP: 4% vs. 8%; *p* = 0.67; Multiplate: 15% vs. 8%; *p* = 0.54). In conclusion, the lower ticagrelor dose provided similar antiplatelet effect to the standard regimen.

Three observational registries aimed to assess the incidence of switching between P2Y_12_ receptor blockers:

### TRANSLATE-ACS

The Longitudinal Assessment of Treatment Patterns and Events after Acute Coronary Syndrome (TRANSLATE-ACS) observational study in 8672 MI patients has reported that P2Y_12_ inhibitor switch occurred in 7.6% of participants (115). The switches were usually de-escalations from more potent agents to clopidogrel mainly due to economic reasons, while escalations from clopidogrel were mainly promoted by ischemic events.

### ATLANTIS-SWITCH

The recently published, prospective, observational, multicenter ATLANTIS-SWITCH study included 571 ACS patients undergoing PCI treated with ticagrelor (45%) or prasugrel (55%) and investigated the frequency and predictors of either switch or drug discontinuation (116). The prevalence of P2Y_12_ antagonist stop was 5.9%, and of switch was 6.7% and it was more frequent in ticagrelor recipients as compared with prasugrel (15.9% vs. 9.2%; *p* = 0.016). The majority of stop/switch choices were prompted by physicians (75%), they did not increase the risk of adverse cardiovascular events and were motivated by one of four identified independent predictors: major surgery, need for oral anticoagulation, TIMI major bleeding, or drug intolerance (116).

### SCOPE

The SCOPE registry investigated the incidence of P2Y_12_ inhibitor switching in 1363 patients undergoing PCI (117). The P2Y_12_ inhibitor switch occurred in 10.5% and was not platelet function based. The authors concluded that de-escalation of antiplatelet treatment from more potent drugs to clopidogrel was an independent predictor of net cerebrovascular event (NACE) defined as a combination of adverse cardiovascular event and any bleeding event (OR 5.3; CI: 2.1–18.2; *p* = 0.04).

### Conclusion of the Chapter

De-escalation strategies with use of platelet function testing seem to be safe.

## Current Place of Platelet Function Testing in Everyday Clinical Practice

The ischemic risk in ACS patients undergoing PCI is relatively high in clopidogrel treated patients due to its heterogenous and unpredictable antiplatelet effect (40, 50). With the common use of more potent antiplatelet agents, increased ischemic risk occurs mainly within first months after ACS, whereas bleeding events are proportional to the duration and intensity of antiplatelet treatment (75, 76). Recently, the idea of de-escalation of antiplatelet therapy was investigated and focused on the net clinical benefit and to minimize the bleeding risk (86, 87). The choice of P2Y_12_ inhibitors offers a chance for individualization of the therapy based on patient characteristics (81, 118). However, the de-escalation trials were powered for minor bleeding events and not for ischemic events. The prolongation studies with P2Y_12_ receptor inhibitors as the DAPT trial or the PEGASUS-TIMI 54 trial indicated benefit for longer treatment with potent drugs as prasugrel or ticagrelor (119, 120). Therefore, in the era of personalized medicine, according to the latest guidelines on myocardial revascularization, platelet function testing guided P2Y_12_ inhibitor de-escalation (e.g., switch from newer more potent drug to clopidogrel after an acute phase) may be considered in ACS patients, particularly those unsuitable for 12-month potent antithrombotic therapy due to the increased bleeding risk (class of recommendation IIb, level of evidence B) (12). Such drug de-escalation could be deliberated highly risky without platelet function testing guidance, especially when we take under consideration very high variability in response to P2Y_12_ receptor inhibitors. In ACS patients undergoing cardiac surgery, platelet function testing is recommended to guide antiplatelet treatment interruption (class of recommendation IIb, level of evidence B), because the preoperative use of P2Y_12_ inhibitors plus aspirin is associated with increased risk of bleeding and mortality (12, 87).

## Conclusions

According to the recent guidelines, platelet function testing use is narrowed to certain clinical scenarios, as P2Y_12_ inhibitor de-escalation and guidance of antiplatelet treatment interruption in ACS patients undergoing cardiac surgery (12). Due to unfavorable results of previous randomized trials its use is not recommended in everyday clinical practice (12, 87). In the course of modern ACS treatment, as directed in the guidelines, a potent P2Y_12_ inhibitor, like prasugrel or ticagrelor, are initiated to prevent ischemic complications, but at the same time taking a risk of increased bleeding. When it comes to a major bleed, a switch to a less potent agent is performed, this time risking possible ischemic complications, leading to a vicious circle. The main goal of the precision-based therapy concept is to provide the right drug in the right dose to fit the needs of an individual patient from the very beginning of the treatment process (29). The physician's choice would then be based on clinical, genetic, cellular and environmental variables. All these data would have to be integrated in an algorithm, as previously proposed (28). The gathered clinical information (e.g., based on the PREDICT score), results of platelet function testing and genetic status (CYP2C19 carrier) could be used to personalize antiplatelet therapy in patients with high-thrombotic or bleeding risk. Moreover, the precision-based antiplatelet therapies are also cost-effective, as this would reduce unnecessary hospitalizations due to either ischemic or bleeding complications. Such a test should be simple, fast, not expensive, well-validated, user-friendly, and platelet function testing fits pretty well to this description.

## Author Contributions

JS-M, MO, and JK contributed conception and design of the manuscript. MO, PA, CE, and AK searched the literature. MO and PA wrote the first draft of the manuscript. JS-M, JK, AK, CE, MP, AT, and CH wrote sections of the manuscript. All authors contributed to manuscript revision, read, and approved the submitted version.

### Conflict of Interest

The authors declare that the research was conducted in the absence of any commercial or financial relationships that could be construed as a potential conflict of interest.

## References

[1] Falk E Nakano M Bentzon JF Finn AV Virmani R. Update on acute coronary syndromes: the pathologists' view. Eur Heart J. (2013) 34:719–28. 10.1093/eurheartj/ehs411 23242196

[2] Davi G Patrono C. Platelet activation and atherothrombosis. N Engl J Med. (2007) 357:2482–94. 10.1056/NEJMra071014 18077812

[3] Siller-Matula JM Lang IM Schoergenhofer C Roest M Jilma B. Interdependence between osteoprotegerin and active von Willebrand factor in long-term cardiovascular mortality prediction in patients undergoing percutaneous coronary intervention. Thromb Haemost. (2017) 117:1730–8. 10.1160/TH17-02-0087 28726980

[4] Varga-Szabo D Pleines I Nieswandt B. Cell adhesion mechanisms in platelets. Arterioscler Thromb Vasc Biol. (2008) 28:403–12. 10.1161/ATVBAHA.107.150474 18174460

[5] Raju NC Eikelboom JW Hirsh J. Platelet ADP-receptor antagonists for cardiovascular disease: past, present and future. Nat Clin Pract Cardiovasc Med. (2008) 5:766–80. 10.1038/ncpcardio1372 18957959

[6] Hechler B Leon C Vial C Vigne P Frelin C Cazenave JP. The P2Y1 receptor is necessary for adenosine 5'-diphosphate-induced platelet aggregation. Blood. (1998) 92:152–9. 10.1182/blood.V92.1.152.413k27_152_159 9639511

[7] Jin J Daniel JL Kunapuli SP. Molecular basis for ADP-induced platelet activation: II. The P2Y1 receptor mediates ADP-induced intracellular calcium mobilization and shape change in platelets. J Biol Chem. (1998) 273:2030–4. 10.1074/jbc.273.4.2030 9442040

[8] Hollopeter G Jantzen H Vincent D Li G England L Ramakrishnan V. Identification of the platelet ADP receptor targeted by antithrombotic drugs. Nature. (2001) 409:202–7. 10.1038/35051599 11196645

[9] Lindemann S Krämer B Seizer P Gawaz M. Platelets, inflammation and atherosclerosis. J Thromb Haemost. (2007) 5:203–11. 10.1111/j.1538-7836.2007.02517.x 17635728

[10] Awtry EH Loscalzo J. Aspirin. Circulation. (2000) 101:1206–18. 10.1161/01.CIR.101.10.1206 10715270

[11] Iakovou I Schmidt T Bonizzoni E Ge L Sangiorgi GM Stankovic G. Incidence, predictors, and outcome of thrombosis after successful implantation of drug-eluting stents. J Am Med Assoc. (2005) 293:2126–30. 10.1001/jama.293.17.2126 15870416

[12] Neumann FJ Sousa-Uva M Ahlsson A Alfonso F Banning AP Benedetto U 2018 ESC/EACTS Guidelines on myocardial revascularization. Eur Heart J. (2019) 40:87–165. 10.1093/eurheartj/ehy394 30165437

[13] Smith J Willis A. Aspirin selectively inhibits prostaglandin production in human platelets. Nat New Biol. (1971) 231:235–7. 10.1038/newbio231235a0 5284361

[14] Ibanez B James S Agewall S Antunes MJ Bucciarelli-Ducci C Bueno H. 2017 ESC Guidelines for the management of acute myocardial infarction in patients presenting with ST-segment elevation: the task Force for the management of acute myocardial infarction in patients presenting with ST-segment elevation of the European Society of Cardiology (ESC). Eur Heart J. (2018) 39:119–77. 10.1093/eurheartj/ehx393 28886621

[15] Roffi M Patrono C Collet JP Mueller C Valgimigli M Andreotti F. 2015 ESC Guidelines for the management of acute coronary syndromes in patients presenting without persistent ST-segment elevation: Task Force for the Management of Acute Coronary Syndromes in Patients Presenting without Persistent ST-Segment Elevation of the European Society of Cardiology (ESC). Eur Heart J. (2016) 37:267–315. 10.1093/eurheartj/ehv320 26320110

[16] Knuuti J Wijns W Saraste A Capodanno D Barbato E Funck-Brentano C. 2019 ESC Guidelines for the diagnosis and management of chronic coronary syndromes. Eur Heart J. (2019). [Epub ahead of print]. 31504439 10.1093/eurheartj/ehz425

[17] Winter MP Schneeweiss T Cremer R Biesinger B Hengstenberg C Prüller F Platelet reactivity patterns in patients treated with dual antiplatelet therapy. Eur J Clin Invest. (2019) 49:e13102 10.1111/eci.13102 PMC6593782 30882911

[18] Ibanez B Vilahur G Badimon JJ Pharmacology of thienopyridines: rationale for dual pathway inhibition. Eur Heart J Suppl. (2006) 8:G3–9. 10.1093/eurheartj/sul047

[19] Adamski P Adamska U Ostrowska M Navarese EP Kubica J. Evaluating current and emerging antithrombotic therapy currently available for the treatment of acute coronary syndrome in geriatric populations. Expert Opin Pharmacother. (2018) 19:1415–25. 10.1080/14656566.2018.1510487 30132731

[20] Siller-Matula JM Petre A Delle-Karth G Huber K Ay C Lordkipanidzé M. Impact of preoperative use of P2Y12 receptor inhibitors on clinical outcomes in cardiac and non-cardiac surgery: a systematic review and meta-analysis. Eur Heart J Acute Cardiovasc Care. (2017) 6:753–70. 10.1177/2048872615585516 25943554

[21] Kubisa MJ Jezewski MP Gasecka A Siller-Matula JM Postuła M. Ticagrelor - toward more efficient platelet inhibition and beyond. Ther Clin Risk Manag. (2018) 14:129–40. 10.2147/TCRM.S152369 29398917 PMC5775739

[22] Adamski P Kozinski M Ostrowska M Fabiszak T Navarese EP Paciorek P. Overview of pleiotropic effects of platelet P2Y12 receptor inhibitors. Thromb Haemost. (2014) 112:224–42. 10.1160/TH13-11-0915 24763899

[23] Hengstenberg C Siller-Matula JM. Shedding light on long-term effects of early antiplatelet strategies after fibrinolytic treatment in STEMI. J Am Coll Cardiol. (2019) 73:2829–31. 10.1016/j.jacc.2019.04.008 31171088

[24] Patti G Pecen L Lucerna M Huber K Rohla M Renda G. Outcomes of anticoagulated patients with atrial fibrillation treated with or without antiplatelet therapy - a pooled analysis from the PREFER in AF and PREFER in AF PROLONGATON registries. Int J Cardiol. (2018) 270:160–6. 10.1016/j.ijcard.2018.06.098 30220376

[25] Schror K Weber AA. Comparative pharmacology of GP IIb/IIIa antagonists. J Thromb Thrombolysis. (2003) 15:71–80. 10.1023/B:THRO.0000003308.63022.8d 14618072

[26] Ostrowska M Adamski P Kozinski M Navarese EP Fabiszak T Grześk G. Off-target effects of glycoprotein IIb/IIIa receptor inhibitors. Cardiol J. (2014) 21:458–64. 10.5603/CJ.a2014.0020 24526503

[27] Siller-Matula JM Trenk D Schrör K Gawaz M Kristensen SD Storey RF EPA (European Platelet Academy). Response variability to P2Y12 receptor inhibitors: expectations and reality. JACC Cardiovasc Interv. (2013) 6:1111–28. 10.1016/j.jcin.2013.06.011 24262612

[28] Siller-Matula JM Trenk D Schrör K Gawaz M Kristensen SD Storey RF How to improve the concept of individualised antiplatelet therapy with P2Y12 receptor inhibitors-is an algorithm the answer? Thromb Haemost. (2015) 113:37–52. 10.1160/TH14-03-0238 25231675

[29] Winter MP Grove EL De Caterina R Gorog DA Ahrens I Geisler T. Advocating cardiovascular precision medicine with P2Y12 receptor inhibitors. Heart J Cardiovasc Pharmacother. (2017) 3:221–34. 10.1093/ehjcvp/pvw044 28204303

[30] Tantry US Bonello L Aradi D Price MJ Jeong YH Angiolillo DJ. Consensus and update on the definition of on-treatment platelet reactivity to adenosine diphosphate associated with ischemia and bleeding. J Am Coll Cardiol. (2013) 62:2261–73. 10.1016/j.jacc.2013.07.101 24076493

[31] Aradi D Kirtane A Bonello L Gurbel PA Tantry US Huber K. Bleeding and stent thrombosis on P2Y12-inhibitors: collaborative analysis on the role of platelet reactivity for risk stratification after percutaneous coronary intervention. Eur Heart J. (2015) 36:1762–71. 10.1093/eurheartj/ehv104 25896078

[32] Kozinski M Ostrowska M Adamski P Sikora J Sikora A Karczmarska-Wodzka A. Which platelet function test best reflects the *in vivo* plasma concentrations of ticagrelor and its active metabolite? The HARMONIC study. Thromb Haemost. (2016) 116:1140–9. 10.1160/TH16-07-0535 27628615

[33] Komosa A Rzymski P Perek B Ropacka-Lesiak M Lesiak M Siller-Matula JM. Platelets redox balance assessment: Current evidence and methodological considerations. Vascul Pharmacol. (2017) 93–95:6–13. 10.1016/j.vph.2017.06.002 28684282

[34] Gurbel PA Bliden KP Hiatt BL O'Connor CM. Clopidogrel for coronary stenting: response variability, drug resistance, and the effect of pretreatment platelet reactivity. Circulation. (2003) 107:2908–13. 10.1161/01.CIR.0000072771.11429.83 12796140

[35] Gurbel PA Bliden KP Hayes KM Yoho JA Herzog WR Tantry US. The relation of dosing to clopidogrel responsiveness and the incidence of high post-treatment platelet aggregation in patients undergoing coronary stenting. J Am Coll Cardiol. (2005) 45:1392–6. 10.1016/j.jacc.2005.01.030 15862408

[36] Angiolillo DJ Fernandez-Ortiz A Bernardo E Ramírez C Sabaté M Bañuelos C. High clopidogrel loading dose during coronary stenting: effects on drug response and interindividual variability. Eur Heart J. (2004) 25:1903–10. 10.1016/j.ehj.2004.07.036 15522469

[37] Mega JL Simon T. Pharmacology of antithrombotic drugs: an assessment of oral antiplatelet and anticoagulant treatments. Lancet. (2015) 386:281–91. 10.1016/S0140-6736(15)60243-4 25777662

[38] Gurbel PA Tantry US. Drug insight: clopidogrel nonresponsiveness. Nat Clin Pract Cardiovasc Med. (2006) 3:387–95. 10.1038/ncpcardio0602 16810174

[39] Gurbel PA Becker RC Mann KG Steinhubl SR Michelson AD. Platelet function monitoring in patients with coronary artery disease. J Am Coll Cardiol. (2007) 50:1822–34. 10.1016/j.jacc.2007.07.051 17980247

[40] Aradi D Komócsi A Vorobcsuk A Rideg O Tokés-Füzesi M Magyarlaki T. Prognostic significance of high on-clopidogrel platelet reactivity after percutaneous coronary intervention: systematic review and meta-analysis. Am Heart J. (2010) 160:543–51. 10.1016/j.ahj.2010.06.004 20826265

[41] Ait-Mokhtara O Bonelloa L Benamarab S Paganelli F High on treatment platelet reactivity. Heart Lung Circ. (2012) 21:12–21. 10.1016/j.hlc.2011.08.069 22000771

[42] Mallouk N Labruyère C Reny JL Chapelle C Piot M Fontana P. Prevalence of poor biological response to clopidogrel: a systematic review. Thromb Haemost. (2012) 107:494–506. 10.1160/TH11-03-0202 22273694

[43] Siller-Matula JM Delle-Karth G Lang IM Neunteufl T Kozinski M Kubica J. Phenotyping vs. genotyping for prediction of clopidogrel efficacy and safety: the PEGASUS-PCI study. J Thromb Haemost. (2012) 10:529–42. 10.1111/j.1538-7836.2012.04639.x 22260716

[44] Navarese EP Verdoia M Schaffer A Suriano P Kozinski M Castriota F. Ischaemic and bleeding complications with new, compared to standard, ADP-antagonist regimens in acute coronary syndromes: a meta–analysis of randomized trials. QJM. (2011) 104:561–9. 10.1093/qjmed/hcr069 21572108

[45] Kubica A Kozinski M Grześk G Goch A Znaczenie kliniczne interakcji miedzy klopidogrelem a inhibitorami pompy protonowej. Kardiol Pol. (2011) 69:610–6. 21678305

[46] Kubica A Kosobucka A Fabiszak T Gorog DA Siller-Matula JM. Assessment of adherence to medication in patients after myocardial infarction treated with percutaneous coronary intervention. Is there a place for newself-reported questionnaires? Curr Med Res Opin. (2019) 35:341–9. 10.1080/03007995.2018.1510385 30091642

[47] Bergmeijer TO Reny JL Pakyz RE Gong L Lewis JP Kim EY. Genome-wide and candidate gene approaches of clopidogrel efficacy using pharmacodynamic and clinical end points-Rationale and design of the International Clopidogrel Pharmacogenomics Consortium (ICPC). Am Heart J. (2018) 198:152–9. 10.1016/j.ahj.2017.12.010 29653637 PMC5903579

[48] Milanowski L Rasul F Gajda SN Eyileten C Siller-Matula JM Postula M. Genetic variability of SRC family kinases and its association with platelet hyperreactivity and clinical outcomes: a systematic review. Curr Pharm Des. (2018) 24:628–40. 10.2174/1381612824666171213105002 29237371

[49] Navarese EP Kolodziejczak M Winter MP Alimohammadi A Lang IM Buffon A. Association of PCSK9 with platelet reactivity in patients with acute coronary syndrome treated with prasugrel or ticagrelor: The PCSK9-REACT study. Int J Cardiol. (2017) 227:644–9. 10.1016/j.ijcard.2016.10.084 27810295

[50] Stone GW Witzenbichler B Weisz G Rinaldi MJ Neumann FJ Metzger DC. Platelet reactivity and clinical outcomes after coronary artery implantation of drug-eluting stents (ADAPT-DES): a prospective multicentre registry study. Lancet. (2013) 382:614–23. 10.1016/S0140-6736(13)61170-8 23890998

[51] Komosa A Siller-Matula JM Lesiak M Michalak M Kowal J Maczynski M. Association between high on-treatment platelet reactivity and occurrence of cerebral ischemic events in patients undergoing percutaneous coronary intervention. Thromb Res. (2016) 138:49–54. 10.1016/j.thromres.2015.12.021 26826508

[52] Parodi G Valenti R Bellandi B Migliorini A Marcucci R Comito V. Comparison of prasugrel and ticagrelor loading doses in ST-segment elevation myocardial infarction patients: RAPID (Rapid Activity of Platelet Inhibitor Drugs) primary PCI study. J Am Coll Cardiol. (2013) 61:1601–6. 10.1016/j.jacc.2013.01.024 23500251

[53] Kubica J Adamski P Ostrowska M Sikora J Kubica JM Sroka WD. Morphine delays and attenuates ticagrelor exposure and action in patients with myocardial infarction: the randomized, double-blind, placebo-controlled IMPRESSION trial. Eur Heart J. (2016) 37:245–52. 10.1093/eurheartj/ehv547 26491112 PMC4712351

[54] Siller-Matula JM Akca B Neunteufl T Maurer G Lang IM Kreiner G. Inter-patient variability of platelet reactivity in patients treated with prasugrel and ticagrelor. Platelets. (2016) 27:373–7. 10.3109/09537104.2015.1095874 26555925

[55] Siller-Matula JM Hintermeier A Kastner J Kreiner G Maurer G Kratochwil C. Distribution of clinical events across platelet aggregation values in all-comers treated with prasugrel and ticagrelor. Vascul Pharmacol. (2016) 79:6–10. 10.1016/j.vph.2016.01.003 26804766

[56] Winter MP Kozinski M Kubica J Aradi D Siller-Matula JM. Personalized antiplatelet therapy with P2Y12 receptor inhibitors: benefits and pitfalls. Postepy Kardiol Interwencyjnej. (2015) 11:259–80. 10.5114/pwki.2015.55596 26677375 PMC4679793

[57] Komosa A Lesiak M Krasinski Z Grygier M Siniawski A Skorupski W. Optimal timing of P2Y12 inhibitor loading in patients undergoing PCI: a meta-analysis. Thromb Haemost. (2019) 119:1000–20. 10.1055/s-0039-1683421 30919382

[58] Lemesle G Schurtz G Bauters C Hamon M. High on-treatment platelet reactivity with ticagrelor versus prasugrel: a systematic review and meta-analysis. J Thromb Haemost. (2015) 13:931–42. 10.1111/jth.12907 25809392

[59] Teng R Mitchell P Butler K. Effect of age and gender on pharmacokinetics and pharmacodynamics of a single ticagrelor dose in healthy individuals. Eur J Clin Pharmacol. (2012) 68:1175–82. 10.1007/s00228-012-1227-4 22367426

[60] Teng R Mitchell PD Butler K. Lack of significant food effect on the pharmacokinetics of ticagrelor in healthy volunteers. J Clin Pharm Ther. (2012) 37:464–8. 10.1111/j.1365-2710.2011.01307.x 21967645

[61] Varenhorst C Eriksson N Johansson Å Barratt BJ Hagstroem E Åkerblom A. Effect of genetic variations on ticagrelor plasma levels and clinical outcomes. Eur Heart J. (2015) 36:1901–12. 10.1093/eurheartj/ehv116 25935875

[62] Kubica J Kubica A Jilma B Adamski P Hobl EL Navarese EP. Impact of morphine on antiplatelet effects of oral P2Y12 receptor inhibitors. Int J Cardiol. (2016) 215:201–8. 10.1016/j.ijcard.2016.04.077 27128531

[63] Sikora J Niezgoda P Baranska M Buszko K Skibinska N Sroka W. METoclopramide Administration as a Strategy to Overcome MORPHine-ticagrelOr Interaction in PatientS with Unstable Angina PectorIS-The METAMORPHOSIS Trial. Thromb Haemost. (2018) 118:2126–33. 10.1055/s-0038-1675605 30453344

[64] Niezgoda P Sikora J Baranska M Sikora A Buszko K Sieminska E. Crushed sublingual versus oral ticagrelor administration strategies in patients with unstable angina. A pharmacokinetic/pharmacodynamic study. Thromb Haemost. (2017) 117:718–26. 10.1160/TH16-08-0670 28203684

[65] Adamski P Sikora J Laskowska E Buszko K Ostrowska M Uminska JM. Comparison of bioavailability and antiplatelet action of ticagrelor in patients with ST-elevation myocardial infarction and non-ST-elevation myocardial infarction: A prospective, observational, single-centre study. PLoS ONE. (2017) 12:e0186013. 10.1371/journal.pone.0186013 29023473 PMC5638327

[66] Adamski P Buszko K Sikora J Niezgoda P Fabiszak T Ostrowska M. Determinants of high platelet reactivity in patients with acute coronary syndromes treated with ticagrelor. Sci Rep. (2019) 9:3924. 10.1038/s41598-019-40628-0 30850677 PMC6408477

[67] Adamski P Buszko K Sikora J Niezgoda P Baranska M Ostrowska M. Metabolism of ticagrelor in patients with acute coronary syndromes. Sci Rep. (2018) 8:11746. 10.1038/s41598-018-29619-9 30082687 PMC6078957

[68] Schoergenhofer C Hobl EL Schellongowski P Heinz G Speidl WS Siller-Matula JM. Clopidogrel in critically Ill patients. Clin Pharmacol Ther. (2018) 103:217–23. 10.1002/cpt.878 28913918 PMC5813104

[69] Uminska JM Ratajczak J Buszko K Sobczak P Sroka W Marszałł MP. Impact of mild therapeutic hypothermia on bioavailability of ticagrelor in patients with acute myocardial infarction after out-of-hospital cardiac arrest. Cardiol J. (2019). [Epub ahead of print]. 30799546 10.5603/CJ.a2019.0024 PMC8079092

[70] Eyileten C Soplinska AM Pordzik J Siller-Matula JM Postuła M. Effectiveness of antiplatelet drugs under therapeutic hypothermia a comprehensive review. Clin. Pharmacol. Ther. (2019) 106:993–1005. 10.1002/cpt.1492 31055838

[71] Prüller F Milke OL Bis L Fruhwald F Scherr D Eller P. Impaired aspirin-mediated platelet function inhibition in resuscitated patients with acute myocardial infarction treated with therapeutic hypothermia: a prospective, observational, non-randomized single-centre study. Ann Intensive Care. (2018) 8:28. 10.1186/s13613-018-0366-x 29468430 PMC5821616

[72] Prüller F Bis L Milke OL Fruhwald F Pätzold S Altmanninger-Sock S. Cangrelor induces more potent platelet inhibition without increasing bleeding in resuscitated patients. J Clin Med. (2018) 7:E442. 10.3390/jcm7110442 30445678 PMC6262477

[73] Siller-Matula JM Specht S Kubica J Alexopoulos D De Caterina R Hobl EL. Abciximab as a bridging strategy to overcome morphine-prasugrel interaction in STEMI patients. Br J Clin Pharmacol. (2016) 82:1343–50. 10.1111/bcp.13053 27366874 PMC5061801

[74] Schoergenhofer C Hobl EL Staudinger T Speidl WS Heinz G Siller-Matula JM. Prasugrel in critically ill patients. Thromb Haemost. (2017) 117:1582–7. 10.1160/TH17-03-0154 28692105 PMC6292180

[75] Wiviott SD Braunwald E McCabe CH Montalescot G Ruzyllo W Gottlieb S. Prasugrel versus clopidogrel in patients with acute coronary syndromes. N Engl J Med. (2007) 357:2001–15. 10.1056/NEJMoa0706482 17982182

[76] Wallentin L Becker RC Budaj A Cannon CP Emanuelsson H Held C. Ticagrelor versus clopidogrel in patients with acute coronary syndromes. N Eng J Med. (2009) 361:1045–57. 10.1056/NEJMoa0904327 19717846

[77] Cuisset T Cayla G Frere C Quilici J Poyet R Gaborit B. Predictive value of post-treatment platelet reactivity for occurrence of post-discharge bleeding after non-ST elevation acute coronary syndrome. Shifting from antiplatelet resistance to bleeding risk assessment? EuroIntervention. (2009) 5:325–9. 10.4244/51 19736156

[78] Sibbing D Schulz S Braun S Morath T Stegherr J Mehilli J. Antiplatelet effects of clopidogrel and bleeding in patients undergoing coronary stent placement. J Thromb Haemost. (2010) 8:250–6. 10.1111/j.1538-7836.2009.03709.x 19943882

[79] Serebruany V Rao SV Silva MA Donovan JL Kannan AO Makarov L. Correlation of inhibition of platelet aggregation after clopidogrel with post discharge bleeding events: assessment by different bleeding classifications. Eur Heart J. (2010) 31:227–35. 10.1093/eurheartj/ehp434 19854728

[80] Patti G Lucerna M Pecen L Siller-Matula JM Cavallari I Kirchhof P. Thromboembolic risk, bleeding outcomes and effect of different antithrombotic strategies in very elderly patients with atrial fibrillation: a sub-analysis from the PREFER in AF (PREvention oF Thromboembolic Events-European Registry in Atrial Fibrillation). J. Am Heart Assoc. (2017) 6:e005657. 10.1161/JAHA.117.005657 28736385 PMC5586290

[81] Renda G Patti G Lang IM Siller-Matula JM Hylek EM Ambrosio G. Thrombotic and hemorrhagic burden in women: gender-related issues in the response to antithrombotic therapies. Int J Cardiol. (2019) 286:198–207. 10.1016/j.ijcard.2019.02.004 30777407

[82] Deharo P Bassez C Bonnet G Pankert M Quilici J Lambert M. Prasugrel versus ticagrelor in acute coronary syndrome: a randomized comparison. Int J Cardiol. (2013) 170:e21–2. 10.1016/j.ijcard.2013.10.043 24231059

[83] Alexopoulos D Stavrou K Koniari I Gkizas V Perperis A Kontoprias K. Ticagrelor vs prasugrel one-month maintenance therapy: impact on platelet reactivity and bleeding events. Thromb Haemost. (2014) 112:551–7. 10.1160/TH14-02-0119 24990396

[84] Sibbing D Steinhubl SR Schulz S Schömig A Kastrati A. Platelet aggregation and its association with stent thrombosis and bleeding in clopidogrel-treated patients: initial evidence of a therapeutic window. J Am Coll Cardiol. (2010) 56:317–8. 10.1016/j.jacc.2010.03.048 20633826

[85] Kubica J Adamski P Buszko K Baranska M Sikora J Marszall MP Platelet inhibition with standard versus lower maintenance dose of ticagrelor early after myocardial infarction (ELECTRA): a randomized, open-label, active-controlled pharmacodynamic and pharmacokinetic study. Eur Heart J Cardiovasc Pharmacother. (2019) 5:139–48. 10.1093/ehjcvp/pvz004 30689800

[86] Cuisset T Deharo P Quilici J Johnson TW Deffarges S Bassez C. Benefit of switching dual antiplatelet therapy after acute coronary syndrome: the TOPIC (timing of platelet inhibition after acute coronary syndrome) randomized study. Eur Heart J. (2017) 38:3070–8. 10.1093/eurheartj/ehx175 28510646

[87] Sibbing D Aradi D Jacobshagen C Gross L Trenk D Geisler T. Guided de-escalation of antiplatelet treatment in patients with acute coronary syndrome undergoing percutaneous coronary intervention (TROPICAL-ACS): a randomised, open-label, multicentre trial. Lancet. (2017) 390:1747–57. 10.1016/S0140-6736(17)32155-4 28855078

[88] Cayla G Cuisset T Silvain J Leclercq F Manzo-Silberman S Saint-Etienne C. Platelet function monitoring to adjust antiplatelet therapy in elderly patients stented for an acute coronary syndrome (ANTARCTIC): an open-label, blinded-endpoint, randomised controlled superiority trial. Lancet. (2016) 388:2015–22. 10.1016/S0140-6736(16)31323-X 27581531

[89] Aradi D Tornyos A Pintér T Vorobcsuk A Kónyi A Faluközy J. Optimizing P2Y12 receptor inhibition in patients with acute coronary syndrome on the basis of platelet function testing: impact of prasugrel and high-dose clopidogrel. J Am Coll Cardiol. (2014) 63:1061–70. 10.1016/j.jacc.2013.12.023 24486281

[90] Christ G Siller-Matula JM Francesconi M Dechant C Grohs K Podczeck-Schweighofer A. Individualising dual antiplatelet therapy after percutaneous coronary intervention: the IDEAL-PCI registry. BMJ Open. (2014) 4:e005781. 10.1136/bmjopen-2014-005781 25361837 PMC4216867

[91] Mayer K Schulz S Bernlochner I Morath T Braun S Hausleiter J. A comparative cohort study on personalised antiplatelet therapy in PCI-treated patients with high on-clopidogrel platelet reactivity. Results of the ISAR-HPR registry. Thromb Haemost. (2014) 112:342–51. 10.1160/TH13-10-0874 24718389

[92] Siller-Matula JM Francesconi M Dechant C Jilma B Maurer G Delle-Karth G. Personalized antiplatelet treatment after percutaneous coronary intervention: the MADONNA study. Int J Cardiol. (2013) 167:2018–23. 10.1016/j.ijcard.2012.05.040 22656044

[93] Kozinski M Obonska K Stankowska K Navarese EP Fabiszak T Stolarek W. Prasugrel overcomes high on-clopidogrel platelet reactivity in the acute phase of acute coronary syndrome and maintains its antiplatelet potency at 30-day follow-up. Cardiol J. (2014) 21:547–56. 10.5603/CJ.a2014.0026 24671900

[94] Trenk D Stone GW Gawaz M Kastrati A Angiolillo DJ Müller U. A randomized trial of prasugrel versus clopidogrel in patients with high platelet reactivity on clopidogrel after elective percutaneous coronary intervention with implantation of drug-eluting stents: results of the TRIGGER-PCI (Testing Platelet Reactivity In Patients Undergoing Elective Stent Placement on Clopidogrel to Guide Alternative Therapy With Prasugrel) study. J Am Coll Cardiol. (2012) 59:2159–64. 10.1016/j.jacc.2012.02.026 22520250

[95] Collet JP Cuisset T Rangé G Cayla G Elhadad S Pouillot C. Bedside monitoring to adjust antiplatelet therapy for coronary stenting. N Engl J Med. (2012) 367:2100–9. 10.1056/NEJMoa1209979 23121439

[96] Price MJ Berger PB Teirstein PS Tanguay JF Angiolillo DJ Spriggs D GRAVITAS Investigators. Standard- vs high-dose clopidogrel based on platelet function testing after percutaneous coronary intervention: the GRAVITAS randomized trial. JAMA. (2011) 305:1097–105. 10.1001/jama.2011.290 21406646

[97] Alexopoulos D Xanthopoulou I Davlouros P Plakomyti TE Panagiotou A Mavronasiou E. Prasugrel overcomes high on-clopidogrel platelet reactivity in chronic coronary artery disease patients more effectively than high dose (150 mg) clopidogrel. Am Heart J. (2011) 162:733–9. 10.1016/j.ahj.2011.07.026 21982667

[98] Alexopoulos D Panagiotou A Xanthopoulou I Komninakis D Kassimis G Davlouros P. Antiplatelet effects of prasugrel vs double clopidogrel in patients on hemodialysis and high on-treatment platelet reactivity. J Thromb Haemost. (2011) 9:2379–85. 10.1111/j.1538-7836.2011.04531.x 21985070

[99] Capranzano P Tamburino C Capodanno D Miccichè E D'Urso L Calvi V. Platelet function profiles in the elderly: Results of a pharmacodynamic study in patients on clopidogrel therapy and effects of switching to prasugrel 5 mg in patients with high platelet reactivity. Thromb Haemost. (2011) 106:1149–57. 10.1160/TH11-05-0346 22011914

[100] Ferreiro JL Ueno M Desai B Capranzano P Capodanno D Angiolillo DJ. Impact of adjunctive cilostazol therapy versus high maintenance dose of clopidogrel in suboptimal responders with diabetes mellitus. Rev Esp Cardiol. (2011) 65:105–6. 10.1016/j.recesp.2011.04.008 21783310

[101] Neubauer H Kaiser AF Endres HG Krüger JC Engelhardt A Lask S. Tailored antiplatelet therapy can overcome clopidogrel and aspirin resistance–the BOchum CLopidogrel and Aspirin Plan (BOCLA-Plan) to improve antiplatelet therapy. BMC Med. (2011) 9:3. 10.1186/1741-7015-9-3 21226927 PMC3033359

[102] Gurbel PA Bliden KP Antonino MJ Stephens G Gretler DD Jurek MM. The effect of elinogrel on high platelet reactivity during dual antiplatelet therapy and the relation to CYP2C19^*^2 genotype: first experience in patients. J Thromb Haemost. (2010) 8:43–53. 10.1111/j.1538-7836.2009.03648.x 19817997

[103] Gurbel PA Bliden KP Butler K Antonino MJ Wei C Teng R Response to ticagrelor in clopidogrel nonresponders and responders and effect of switching therapies: the VA study. Circulation. (2010) 121:1188–99. 10.1161/CIRCULATIONAHA.109.919456 20194878

[104] Valgimigli M Campo G de Cesare N Meliga E Vranckx P Furgieri A Intensifying platelet inhibition with tirofiban in poor responders to aspirin, clopidogrel, or both agents undergoing elective coronary intervention: results from the double-blind, prospective, randomized Tailoring Treatment with Tirofiban in Patients Showing Resistance to Aspirin and/or Resistance to Clopidogrel study. Circulation. (2009) 119:3215–22. 10.1161/CIRCULATIONAHA.108.833236 19528337

[105] Jeong YH Lee SW Choi BR Kim IS Seo MK Kwak CH. Randomized comparison of adjunctive cilostazol versus high maintenance dose clopidogrel in patients with high post-treatment platelet reactivity: results of the ACCEL-RESISTANCE (Adjunctive Cilostazol Versus High Maintenance Dose Clopidogrel in Patients With Clopidogrel Resistance) randomized study. J Am Coll Cardiol. (2009) 53:1101–9. 10.1016/j.jacc.2008.12.025 19324253

[106] Bonello L Camoin-Jau L Armero S Com O Arques S Burignat-Bonello C. Tailored clopidogrel loading dose according to platelet reactivity monitoring to prevent acute and subacute stent thrombosis. Am J Cardiol. (2009) 103:5–10. 10.1016/j.amjcard.2008.08.048 19101221

[107] Bonello L Camoin-Jau L Arques S Boyer C Panagides D Wittenberg O. Adjusted clopidogrel loading doses according to vasodilator-stimulated phosphoprotein phosphorylation index decrease rate of major adverse cardiovascular events in patients with clopidogrel resistance: a multicenter randomized prospective study. J Am Coll Cardiol. (2008) 51:1404–11. 10.1016/j.jacc.2007.12.044 18387444

[108] Aleil B Jacquemin L De Poli F Zaehringer M Collet JP Montalescot G. Clopidogrel 150 mg/day to overcome low responsiveness in patients undergoing elective percutaneous coronary intervention: results from the VASP-02 (Vasodilator-Stimulated Phosphoprotein-02) randomized study. JACC Cardiovasc Interv. (2008) 1:631–8. 10.1016/j.jcin.2008.09.004 19463377

[109] Trenk D Hochholzer W Muller B Stratz C Valina CM Schmiebusch P. Antiplatelet response to the 150-mg maintenance dose of clopidogrel in patients with insufficient platelet inhibition after clopidogrel loading for elective coronary stent placement. EuroIntervention. (2008) 4:214–21. 10.4244/EIJV4I2A39 19110786

[110] Cuisset T Frere C Quilici J Morange PE Mouret JP Bali L. Glycoprotein IIb/IIIa inhibitors improve outcome after coronary stenting in clopidogrel nonresponders: a prospective, randomized study. J Am Coll Cardiol Intv. (2008) 1:649–53. 10.1016/j.jcin.2008.08.018 19463379

[111] Matetzky S Fefer P Shenkman B Varon D Savion N Hod H. Effectiveness of reloading to overcome clopidogrel nonresponsiveness in patients with acute myocardial infarction. Am J Cardiol. (2008) 102:524–9. 10.1016/j.amjcard.2008.04.028 18721506

[112] Neubauer H Lask S Engelhardt A Mügge A. How to optimise clopidogrel therapy? Reducing the low-response incidence by aggregometry-guided therapy modification. Thromb Haemost. (2008) 99:357–62. 10.1160/TH07-10-0624 18278186

[113] Wang XD Zhang DF Zhuang SW Lai Y. Modifying clopidogrel maintenance doses according to vasodilator-stimulated phosphoprotein phosphorylation index improves clinical outcome in patients with clopidogrel resistance. Clin Cardiol. (2011) 34:332–8. 10.1002/clc.20884 21538380 PMC6652726

[114] Ari H Ozkan H Karacinar A Ari S Koca V Bozat T. The EFFect of hIgh-dose ClopIdogrel treatmENT in patients with clopidogrel resistance (the EFFICIENT trial). Int J Cardiol. (2012) 157:374–80. 10.1016/j.ijcard.2010.12.083 21239075

[115] Zettler ME Peterson ED McCoy LA Effron MB Anstrom KJ Henry TD. Switching of adenosine diphosphate receptor inhibitor after hospital discharge among myocardial infarction patients: Insights from the Treatment with Adenosine Diphosphate Receptor Inhibitors: Longitudinal Assessment of Treatment Patterns and Events after Acute Coronary Syndrome (TRANSLATE-ACS) observational study. Am Heart J. (2017) 183:62–8. 10.1016/j.ahj.2016.10.006 27979043

[116] Winter MP von Lewinski D Wallner M Prüller F Kolesnik E Hengstenberg C Incidence, predictors, and prognosis of premature discontinuation or switch of prasugrel or ticagrelor: the ATLANTIS - SWITCH study. Sci Rep. (2019) 9:8194 10.1038/s41598-019-44673-7 31160687 PMC6547711

[117] De Luca L D'Ascenzo F Musumeci G Saia F Parodi G Varbella F. Incidence and outcome of switching of oral platelet P2Y12 receptor inhibitors in patients with acute coronary syndromes undergoing percutaneous coronary intervention: the SCOPE registry. EuroIntervention. (2017) 13:459–66. 10.4244/EIJ-D-17-00092 28374678

[118] Gasecka A Konwerski M Pordzik J Soplinska A Filipiak KJ Siller-Matula JM. Switching between P2Y12 antagonists - From bench to bedside. Vascul Pharmacol. (2019) 115:1–12. 10.1016/j.vph.2019.01.003 30685502

[119] Mauri L Kereiakes DJ Yeh RW Driscoll-Shempp P Cutlip DE Steg PG Twelve or 30 months of dual antiplatelet therapy after drug-eluting stents. N Engl J Med. (2014) 371:2155–66. 10.1056/NEJMoa1409312 25399658 PMC4481318

[120] Bonaca MP Bhatt DL Cohen M Steg PG Storey RF Jensen EC Long-term use of ticagrelor in patients with prior MI. N Eng J Med. (2015) 372:1791–800. 10.1056/NEJMoa1500857 25773268

